# Simian immunodeficiency virus-Vpx as an adjuvant for integrase defective lentiviral vector-based vaccines

**DOI:** 10.1186/1742-4690-9-S2-P293

**Published:** 2012-09-13

**Authors:** M Blasi, A Rossi, Z Michelini, P Leone, G Perretta, A Cimarelli, ME Klotman, D Negri, A Cara

**Affiliations:** 1 Duke University School of Medicine, Durham, NC, USA; 2 Istituto Superiore di Sanita', Roma, Italy; 3 Istituto di Biologia Cellulare e Neurobiologia-CNR ENEA-Casaccia, Roma, Italy; 4 Ecole Normale Superieure de Lyon, Lyon, FL, France

## Background

Integrase defective lentiviral vectors (IDLV) represent a promising delivery system for immunization purposes. Human dendritic cells (DC) are the main cell types mediating the immune response and are readily transduced by IDLV, allowing effective triggering of in vitro expansion of antigen-specific primed CD8+ T cells. However, DC transduction efficiency is hindered by the presence of SAMHD1 restriction factor, which inhibits viral DNA synthesis.

## Methods

IDLV expressing Flu-M1 containing simian immunodeficiency virus (SIV)-Vpx was produced and titred on 293T by standard methods. Monocytes from HLA-A*0201 and M1-positive selected donors were differentiated into DC and transduced with IDLV-M1/Vpx and control IDLV/M1 or left untreated. IDLV-transduced DC were co-cultured with autologous PBMC and the expansion of M1-specific CD8+ T cells was analysed by pentamer staining and IFNγ ELISPOT

## Results

The addition of the SIV-Vpx protein during IDLV preparation resulted in a striking improvement of IDLV transduction of human DC, thus increasing the ability of IDLV-transduced DC to act as functional antigen presenting cells, as evaluated by pentamer staining and IFNγ ELISPOT, in the absence of vector integration. Importantly, the presence of SIV-Vpx allows for the use of lower amount of input vector preparation, further improving the safety profile of IDLV.

## Conclusion

These results have important implications for the development of vaccine strategies based on the use of IDLV as a novel, safe and efficient delivery system.

